# Ten important lessons we have learned as pathology bloggers

**DOI:** 10.4103/2153-3539.70709

**Published:** 2010-10-01

**Authors:** Keith J. Kaplan, Bruce A. Friedman, Mark D. Pool

**Affiliations:** Department of Pathology, Carolinas Pathology Group, Charlotte, NC; 1 Department of Pathology, University of Michigan Medical School, Ann Arbor, MI; 2 Department of Pathology, Rush Medical College, Chicago, IL

A blog (an abridgment of the term ‘web log’) is a type of website or part of a website. Blogs are usually maintained by an individual with regular entries of commentary, descriptions of events, or other material such as graphics or videos.[1,2] Entries are commonly displayed in reverse chronological order. ‘Blog’ can also be used as a verb, meaning to maintain or add content to a blog.

Many blogs provide commentary or news on a particular subject; others function as more personal online diaries. A typical blog combines text, images and links to other blogs, web pages, and other media related to its topic. The ability of readers to leave comments in an interactive format is an important part of many blogs. Most blogs are primarily textual, although some focus on art (Art blog), photographs (photoblog), videos (Video blogging), music (MP3 blog), and audio (podcasting). Microblogging is another type of blogging, featuring very short posts.

As three active bloggers in the fields of pathology, laboratory medicine, and pathology informatics, we thought that it would be useful for us to share the lessons that we have learned in our blogging endeavors.

**Table:** 

Blog	Author	URL	Hosting service	Funding
Digital pathology blog	KJK	http://www.tissuepathology.com	Typepad	Sponsorships
Lab soft news	BAF	http://labsoftnews.typepad.com	Typepad	Sponsorships
The daily sign-out	MDP	http://pathlabmed.typepad.com	Typepad	None

***Lesson 1* - Blogging as professionals**: All professional communication in this information technology era can be viewed as a continuum, with informal office and hallway conversations representing one end of the spectrum and also formal peer-reviewed publications (e.g., journal articles) marking the other end. Blogging fits roughly into the middle of this arc, with some degree of overlap. For example, occasionally our stimulating hallway or email conversations that become blog notes evolve into more formal articles. This communication, in fact, is an example of this latter scenario. We define ourselves as ‘embedded blogging professionals’, which is to say that as trained pathologists we orient most of our blog notes to fellow laboratorians and pathology professionals, actively working in our field. This distinguishes our endeavors from ‘social bloggers’ who write about their passions on topics generally remote from their professional expertise. We also distinguish ourselves from professional (non-pathologist) journalists who often acquire their ‘content’ largely in the context of writing a particular story.

***Lesson 2* - Transparency of the blogger**: Creating a blog is unlike any other form of publishing or communication. There are no intermediaries, no journal editors, and no proximate critics. Bloggers are on your own in the blogosphere, where blogging is a transaction only between you and the readers. With web 2.0 tools most ‘netizens’ (i.e., citizens on the web) now have the ability to inexpensively, quickly, and efficiently publish their ideas and opinions online for a global audience. In so doing, bloggers *disintermediate* layers of editors, authorities, and interlocutors who may have created *information friction*, while providing their functions or services (i.e., selection and editing of ideas). Therefore, as a blogger, one’s thoughts, ideas, images, or questions can ricochet within minutes across the world and be viewed, analyzed, and criticized by a myriad of online readers. One of the most important lessons we have learned about blogging is that the exposed blogger must become accustomed to a new level of transparency when publishing material. While we tend to believe that our pathology blogs are professional and somewhat academic in nature, all published blog notes represent non-peer reviewed personal insights and opinions, reflecting the core values and ideas of the blogger.

***Lesson 3* - Blogging is competitive**: Bloggers rise or fall based on the value and worth of what they have to say. Blogging is competitive. While blog readers do not pay in monetary terms to read your blogs, they ‘pay’ with the very precious commodity of their time. The success of a blogger can be measured almost entirely on the number of readers their notes attract, although the personal satisfaction of blogging should not be discounted. For a professional pathology blog, the target audience is generally peer professionals including health system and corporate executives. However, as pathology bloggers we find that we also provide an opportunity for informed healthcare consumers (e.g., vendors, perhaps patients) to learn more about this field.

One of the temptations encountered when launching a blog is to spend more time trying to emulate other bloggers in the field rather than establishing one’s own voice and style. While there are many lessons to be learned from other blogs, the key to our success was to develop a strong blogging identity or *persona* of our own. Readers are an insightful bunch and will quickly find out if you are not being genuine. It is also difficult to sustain writing in a consistent voice and personality that is not your own. We have found that if you can be yourself you will last a lot longer as a blogger. When you chop and change your writing to emulate and/or please others, you may end up disillusioning the people that make your blog what it is (i.e., your readers).

***Lesson 4* - Blogging links and comments**: While there are no hard-and-fast rules to blogging, the professional blogger has to be mindful and should emulate the profession as a whole. They can still push the boundaries and need not be afraid to make mistakes, which will undoubtedly occur. However, they need to be cognizant of what readers hope to derive from the blog postings. Blogging, it can be argued, is not so much about writing, as it is about linking and commenting. Most bloggers find content online or elsewhere, share that content with their readers, and comment upon it, often through links or re-posted material. Most of our daily postings are of this nature.

Although there are estimated to be well over a billion Internet users worldwide, there are *only* 55 million users (5%) who have blogs. Of these, there are 1.6 million postings per day; because some people post multiple times per day (*only 0.1% of users post daily*). Web user participation often more-or-less follows a 90–9–1 rule: 90% of the users are lurkers (i.e., readers who observe, but do not contribute); 9% of the users contribute from time-to-time, but other priorities dominate their time; 1% of the users participate a lot and account for most contributions. Blogs have perhaps the lowest rate of participation and inequality that characterizes most online communities. With blogs, the rule is more like 95-5-0.1. As an example, we have posted more than two thousand blog notes, however, we have received only around two hundred comments. Although most of our posts are technology, pathology, and clinical laboratory-related and may not necessarily stimulate much comment or commentary, the point is that blogging is a ‘do it yourself’ labor of love. Bloggers sometimes feel like they spend a lot of time ‘talking to themselves’. For the first year or so, we found ourselves asking the question: *Is there anyone out there?*

***Lesson 5* - Find your niche**: Pathology blogging should not be tackled unless you are able to write quickly, regularly, reasonably well, and are self-motivated. A successful blog must provide value. When starting out, one needs to determine what value this blog will give readers. Targeting a niche around more tightly focused topics will better help to define your blog to your readers.

***Lesson 6* - Blogging fosters connectedness**: Blogging fulfils our wish to network with pathology colleagues, other clinical colleagues, and non-physicians seeking information. We have learned through blogging that our activities foster connectedness and may even influence the behavior of others. We have become increasingly more aware of the power of social networks.

**Lesson 7 - Blogs can be creative**: A blogger is a writer and the purpose of the ‘writer’ is to be read as Samuel Johnson has said. Would anyone bother blogging if no one read his or her blogs? Otherwise, this activity is just electronic journaling. What attracts us about blogging are the challenges imposed when creating a blog, such as the distillation of thought, reaction, experience, and opinion into a concise, but incisive missive that someone might find interesting, provocative, and useful. We are learning to refine our own opinions, to be more discriminating consumers of information and continue to develop our own voice. A blogger is also a critic. We often react to something that we have read, viewed or overheard. By blogging about these issues, we feel a sense of responsibility to express thoughtful and reasoned opinions as well as our own doubts, uncertainties or ignorance. We have also enjoyed writing mini-case reports or stories as an effort to capture the immediacy of sharing an interesting case with a colleague as well as a few facts that were discovered in the process of making a diagnosis and learning about a diagnostic entity.

***Lesson 8* - Blogging requires discipline**: Regular blogging imposes a discipline to write and produce notes regularly and to stay engaged with the field. Unlike the sporadic, episodic nature of writing for research where there are rushes to meet abstract or grant application deadlines, blogging is entirely self-directed. Once you get started, blogging regularly becomes part of the bloggers daily routine.

***Lesson 9* - Bloggers are filters**: One of the greatest values in pathology blogging is being forced to keep abreast of new developments in the field. In essence, the blogger functions as a filter of key information for others in the field, and for consumers who turn to the Internet for advice. With the expertise to filter out key information in the field through one’s blogs, the professional blogger provides a service when sharing their message with others.

***Lesson 10* - Benefits from blogging**: Most information on the web is free, and much of this content gets posted online by individuals with no known identity, or even credibility. The value of a professional blogger is to create an identity in the blogosphere of being a knowledgeable observer and interpreter of relevant issues. Hopefully one’s blogged ideas and opinions will become widely known and valued by your veteran readers. Also, on the web as the saying goes, based on a classic New Yorker cartoon, ‘no one knows if you are a dog’. The lessons derived from these sayings are that it is possible to monetize a blog, but generally speaking, the value of a professional blogger is to create an identity in the blogosphere of being a knowledgeable observer and interpreter of relevant issues. Remunerative activities can flow from this web-established identity, such as, consulting, either from a business perspective or as an expert in surgical pathology. The take-home lesson of the ‘dog’ anecdote is that you frequently start your blogging career as a relative unknown — in essence, you create your own web persona based on the quality of your notes, in a highly competitive manner. They may even approach you at social gatherings and while they may feel that they know you intimately, you may have never met them before.

In the final analysis, the greatest value in pathology blogging is forcing yourself to keep abreast of new developments in the field. In essence, you are functioning as a filter for others in the field and for consumers who turn to Dr. Google for advice, so that they can become more efficient and effective healthcare consumers. By turning yourself into such a filter, you become a much more effective and efficient professional working in the field.

## References

[1] http://www.rebeccablood.net/essays/weblog_history.html.

[2] Moore BE (2008). The blog phenomenon hits pathology. Adv Anat Pathol.

